# Comparative study of striatum GABA concentrations and magnetic resonance spectroscopic imaging in Parkinson's disease monkeys

**DOI:** 10.1186/s12868-019-0522-8

**Published:** 2019-08-08

**Authors:** Lixuan Huang, Yande Ren, Zisan Zeng, Hao Ren, Shaojun Li, Shengnan He, Fan He, Xiangrong Li

**Affiliations:** 1 Department of Magnetic Resonance Imaging, The First People’s Hospital of Nanning, Nanning, 530022 Guangxi Province China; 2 Department of Radiology, The Affiliated Hospital of Qingdao University, Qingdao, 266003 Shandong Province China; 3 Department of Radiology, The First Affiliated Hospital of Guangxi Medical University, No. 6, Shuangyong Road, Nanning, 530021 Guangxi Province China; 4 Department of Radiology, Guangxi Medical University Kaiyuan Langdong Hospital, Nanning, 530000 Guangxi Province China; 5 Department of Toxicology, School of Public Health, Guangxi Medical University, Nanning, 530021 Guangxi Province China; 6 Department of Control of Occupational Hazards, Yongzhou Disease Prevention and Control Center, Yongzhou, 425000 Hunan Province China; 7 Department of Radiology, Guangzhou Women and Children’s Medical Center, Guangzhou, 510623 Guangdong Province China

**Keywords:** Magnetic resonance spectroscopy, Parkinson's disease, Striatum, γ-Aminobutyric acid

## Abstract

**Background:**

Parkinson's disease is a progressive degenerative nervous system disease. Recent studies have shown that secondary changes in the GABA system play directly affect the pathogenesis of PD. There is still much debate about GABA concentrations because currently, GABA concentrations in the brain tissue are obtained indirectly by measuring its concentration in the blood and cerebrospinal fluid. These results are unreliable. Magnetic resonance spectroscopy (MRS) is the only noninvasive method for evaluating the concentration of metabolites in living brain tissue and has been widely applied in research and clinical practice. In addition, combining MEGA-PRESS technology with LCModel software for quantitative GABA measurements is largely recognized. At present, the PD monkeys model in primates has been increasingly proficient. Primates are more similar to humans in terms of brain structure and function than other animals. However, 3.0 T MRS studies involving the PD monkey model to measure metabolites in living subjects with PD are still rare. The study was performed at 3.0 T MRI with control monkeys and PD monkeys that were injected methyl-phenyl-tetrahydropyridine (MPTP) in one side of common carotid artery before and 3 months after successful model establishment to measure GABA concentrations in the bilateral striatum. Behavioral observations were performed for all animals, and the behavioral score was recorded. After 3 months, the GABA concentration in the bilateral striatum was measured in both groups by high-performance liquid chromatography (HPLC). The data obtained from magnetic resonance spectroscopy (MRS) were compared with the actual measured GABA concentrations in tissues isolated from the corresponding regions, and their correlations with the behavior score were analyzed. The research objectives are to investigate the changes of γ-aminobutyric acid (GABA) concentration in the bilateral striatum of monkeys with Parkinson's disease (PD) and the value of quantitatively measuring its concentration by noninvasive 3.0 T spectroscopy.

**Results:**

(1) The MRS results showed that the GABA concentration in the injured side of the striatum of the PD monkeys was higher than in the contralateral side, but the difference was not statistically significant (P = 0.154). Compared with that the blank control group, the GABA concentration in the striatum of the PD monkeys increased, but there was no difference between the groups (P = 0.381; P = 0.425). (2) The GABA concentration that determined from the isolated specimens by HPLC in the injured side of the striatum of the PD monkeys was significantly higher than that in the contralateral side (P < 0.01). Compared with the blank control group, the PD monkeys had higher GABA concentrations in both sides of the striatum, and there was a significant difference in the lesion side (P = 0.004), while there was a non-significant difference in the contralateral side (P = 0.475). (3) The mean GABA concentration in the injured striatum of PD monkeys determined by MRS was not significantly correlated with the behavioral score (r = 0.146, P = 0.688). The mean GABA concentration in the injured striatum determined from the isolated specimens was positively correlated with the behavioral score in the same period (r = 0.444, P = 0.038).

**Conclusion:**

The GABA concentration in the injured striatum of PD monkeys is increased and positively correlated with behavioral changes. Validity of noninvasive 3.0 T MRS to detect PD neurotransmitter changes is limited.

## Background

Parkinson's disease is a progressive degenerative nervous system disease and is often accompanied by symptoms such as resting tremor, muscle stiffness, bradykinesia, and posture balance disorder. The pathology is mainly the degeneration of nigrostriatal dopaminergic neurons, which leads to the loss of dopamine production by neuronal projection of the substantia nigra to the dorsal striatum, thereby reducing the normal inhibition of GABA ergic neuron activity [1]. Conventional positron emission tomography (PET) and magnetic resonance imaging (MRI) techniques can show early brain function and structural changes in PD patients [2], such as pons and medullary atrophy, and neuronal loss in this region. However, most studies suggest that nigrostriatal structure does not change [3].

GABA is a amino acid neurotransmitter located in the central nervous system and is mainly generated by the decarboxylation of glutamate (Glu) by glutamic acid decarboxylase (GAD). It binds to GABA receptor and induces a strong biological effect to inhibit neuronal excitability, causing a series of neurodegenerative diseases such as PD. Recent studies have shown that secondary changes in the GABA system play an important and direct role in the pathogenesis of PD [4], and the concentration of GABA can be measured. Yao et al. [5] used HPLC to measure the content of GABA in the cortex of PD rats. It was found that the concentration of GABA in the cortex of the side of 6-hydroxyDA (6-OHDA) injected in the PD rats was higher than in the control side and the control group. This finding is consistent with autopsy results showing increased GABA concentrations in the striatum of PD patient [6]. However, many researchers found the opposite result. Tong et al. [7] found that the plasma GABA concentration was decreased in PD patients compared with healthy controls. Yuan et al. [8] found that the glutamate, aspartic acid and GABA levels in the plasma were significantly lower in early PD patients than in the control group. There is still much debate about the GABA concentrations. Moreover, GABA concentrations in brain tissue is obtained indirectly by measuring its concentration in blood and cerebrospinal fluid, the results are unreliable, and it is difficult to directly or truly measure the concentration in brain tissue [9]. As the only noninvasive method for evaluating the concentration of metabolites in living brain tissue, magnetic resonance spectroscopy (MRS) has been used in human brain research since the 1980s and has been widely used in scientific research and clinical practice. Chassain et al. [1] used MPTP to establish a PD mouse model. By using the 1H-MRS technique, it was observed that the GABA content in the lenticular nucleus was increased. Similarly, some scholars have found that the GABA/Glu ratio in the lenticular nucleus is four times higher than that in the cortical area [6].

Due to the low content of GABA, it is difficult to measure the GABA content via conventional MRS sequences; this measurement has special requirements in terms of spectral editing technology, equipment and field strength. Henry et al. [10] optimized the J-modulation differential technique to create a special spectral editing technique that eliminates the effects of macromolecular signals and is more accurate than previous differential techniques. The LCModel software automates phase correction and baseline correction of data directly outputs the content of metabolites; this software has been adapted for spectra obtained from 1.5 T and 3.0 T magnetic instruments [11]. Combining MEGA-PRESS technology with LCModel software for quantitative GABA measurements has been recognized by the academic community. Scholars have verified the reliability of the method through model experiments [12]. At present, most MRS studies of GABA concentrations use a magnetic field of more than 4 T. Öz et al. [6] detected 10 metabolites in the substantia nigra using 4 T MRS and Emir et al. [3] detected 15 metabolites in the substantia nigra by 7 T MRS. In animal experiments, 9.4 T and 14.1 T have been used [1, 13, 14]; however, relevant research on 3.0 T equipment is rare. Increasing the field strength can increase the sensitivity for detecting metabolites, improving the spectral signal-to-noise ratio and the spatial resolution. However, most of the current clinical applications are based on 1.5 T or 3.0 T. The ability to use 1.5 T and 3.0 T MRI for accurate quantification of GABA levels in the human brain and for successful applications in clinical practice would be of great significance.

The purpose of this study was to compare the GABA concentration in the striatum measured by the 3.0 T MRI MEGA-PRESS spectral editing sequence combined with LCModel software and HPLC with the cynomolgus monkey as an experimental animal, to investigate the changes in GABA concentration in Parkinson's striatum and to evaluate the value of 3.0 T noninvasive spectroscopy for quantitative measurement of GABA concentrations.

## Results

### Postoperative motor score

One week after the model was successfully established, limb muscle stiffness began to appear on the side contralateral to the MPTP injury of the PD monkeys (6/6). In addition, typical PD symptoms, such as limb postural tremor and spontaneous body rotation were sometimes found on the MPTP lesion side. The lowest score was 6 and the highest score was 12.5; the scores remained constant. The limbs of the MPTP injury side did not show symptoms such as muscle stiffness and tremor (Table 1).

**Table 1:** Motor score results for the unilateral PD monkey model

PD model monkey group	Weekly motor score						
	Before model	1 week	3 weeks	5 weeks	7 weeks	9 weeks	11 weeks
M1	0	12	12	12	12	12	11
M2	0	6	8	7.5	7.5	7.5	7
M3	0	6	6.5	6.5	7.5	7.5	7.5
M4	0	8.5	10.5	10.5	9.5	9.5	9.5
M5	0	6	7	8	8	7	8
M6	0	12	12.5	12.5	10	10	10

### Apomorphine-induced experiment

Four weeks after the model was successfully established, the PD monkeys (6/6) showed stable symptoms and were given intramuscular APO. Ten minutes after the injection, a rapid onset of symptoms began to appear on the side of the the MPTP injury and the symptoms accelerated with stimulation, reaching 8 laps/min. This value remained stable for 8 to 12 weeks (Table 2).

**Table 2:** Mean rotations in the unilateral PD monkey model before and after apomorphine induction (rotations/min)

PD model monkey group	4 weeks		8 weeks		12 weeks	
	Before induction	After induction	Before induction	After induction	Before induction	After induction
M1	0	2.3	0.4	3	0.4	2.7
M2	0.1	6.7	0.6	6.5	0.6	7.6
M3	0.2	3.5	0.4	3.7	0.3	4
M4	0.2	4.3	0.3	4.6	0.5	5.2
M5	0	2.6	0.2	3.1	0.6	3.2
M6	0	2.9	0.3	3.3	0.2	3.1

### MRS and isolated tissue GABA concentration

(1) As seen from the MRS results in Table 3, the GABA concentration in the striatum of the injured side of the PD monkey was higher than in the contralateral side, but the difference was not.

**Table 3:** Comparison of GABA concentrations (µmol/L) in the two sides of the striatum before and after the PD model was established by MRS (mean ± SD)

	Before model	After model	*P* value
Left side (affected side)	0.356 ± 0.123	0.405 ± 0.161	0.381
Right side (healthy side)	0.266 ± 0.114	0.323 ± 0.947	0.425
*P* values	*P* = 0.078	*P* = 0.154	

statistically significant (P = 0.154). Compared with that in the blank control group, the GABA concentration in the striatum of the PD monkeys increased, but the increase was not statistically significant (P = 0.381; P = 0.425). (2) The GABA concentration that determined from the isolated specimens by HPLC in the injured side of the striatum of the PD monkeys was significantly higher than in the contralateral side (P < 0.01). Compared with the blank control group, the PD monkeys had higher GABA concentrations in both sides of the striatum; the difference was significant in the lesion side (P = 0.004) but not the contralateral side (P = 0.475) (Table 4). (3) The mean GABA concentration in the injured striatum of PD monkeys measured by MRS was not significantly correlated with the behavioral score (r = 0.146, P = 0.688). The mean GABA concentration in the injured striatum obtained from the isolated specimens was positively correlated with the behavioral score in the same period (r = 0.444, P = 0.038).

**Table 4:** Comparison of GABA concentrations (μmol/g) in the isolated striatum after the PD model was established and in the control group (mean ± SD)

	After model	Control group	*P* values
Left side (affected side)	2.381 ± 0.382	1.485 ± 0.259	P = 0.004
Right side (healthy side)	1.436 ± 0.210	1.540 ± 0.219	P = 0.475
*P* values	*P* < 0.01	*P* = 0.757	

## Discussion

MPTP is oxidized to produce l-methyl-4-phenylpyridinium (MPP+ ), which has neurotoxic effects and destroys dopamine production channels, resulting in PD [15]. It is believed that MPTP can induce severe Parkinson's syndrome in only 3 days, which is contrary to the slow degeneration and progression of PD. According to the Braak stage, the typical motor symptoms appeared with stage 3–4 lesions and the clinical symptoms were significantly different. When clinical symptoms appear in PD patients, the loss of substantia nigra neurons has reached 50–80% [16]. In this study, the unilateral MPTP lesions in PD monkeys were caused by acute injury, which should be further explored relative to the pathological stage of PD patients. This study used a comprehensive motor score scale, APO-induced rotation test, and disappearance of dopaminergic neurons with tyrosine hydroxylase (TH)-positive staining in the substantia nigra for evaluating the success of PD modeling.

The metabolites detectable by noninvasive MRS include *N*-acetylaspartate (NAA), creatine (Cr), choline (Cho), lactate (Lac), myo-inositol (MI), glutamate (GIu), GABA, and glutamine (Gln). The main studies related to PD found changes in GABA, Cho, NAA, and Cr. As early as 1950, GABA was first discovered in mammalian brain extracts and the GABA brain content was extremely low, approximately 5–10 mmol/L [17]. In our previous study, we found that the GABA concentration in the striatum of normal people decreased with age and that the GABA concentration in the striatum increased in PD patients.

In a study measuring GABA concentrations in the PD striatum, Chassain et al. [1] used MPTP to establish a rat model of PD andfound through using the 1H-MRS technique that the GABA content in the lenticular nucleus was increased. Coune et al. [14] used a 14.1 T device for MRS scans to measure the striatum of PD rats and found that the GABA concentration in the injured side was significantly higher than that in the normal side. The authors believed that before the complete neurodegeneration of the substantia nigra, MRS can measure the change in the striatum GABA level which can be used as a sensitive biomarker for PD diagnoses. With the improvement in field strength, Emir et al. [3] used 7.0 T MRS technology to study patients with mild to moderate PD. LCModel was used to measure various metabolites, including GABA, NAA, Cho, and Cr, in the pons, caudate nucleus and substantia nigra. The results showed that the GABA content in the pons, lenticular and putamen of patients with PD significantly increased. Terpstra et al. [6] used 4 T MRS technology to measure the concentration of more than 10 metabolites, including GABA, in patients with PD. The GABA/Glu ratio in the lenticular nucleus was 4 times higher than that in the cortical area. These findings are consistent with PD and autopsy results, the GABA concentration in the lenticular nucleus is increased with PD [4]. Similarly, in this study HPLC was used to measure the GABA concentration in the left striatum of PD monkeys. It was found that the GABA concentration in the left striatum of the PD monkeys was higher than that in the contralateral side and that in the control group, there was a significant difference, which further confirmed that the increase in GABA concentration in the PD striatum was closely related to the pathogenesis of PD.

At present, there are two main methods for accurately measuring GABA concentrations in vivo using MRS technology: (1) Using different spectral editing methods, such as two-dimensional J resolved spectroscopy, the chemical shift correlation spectrum, the magnetization transfer method, the multiple-quantum filter (MQF), and the J-modulation differential technique, to separate GABA at δ 3.0 ppm from the background of macromolecules and to determine the total creatine/high-energy phosphate (Cr/PCr) value; (2) Analysis of GABA and other metabolites using a special software package (LCModel and MRUI are commonly used in research currently) to provide the concentration [6].

However, this study used the 3.0 T MRI MEGA-PRESS spectral editing sequence combined with LCModel software to measure the GABA concentration in the bilateral striatum of PD monkeys. Compared with the healthy side and the control group, the lesion side had higher striatum GABA concentrations, but the difference was not statistically significant. This finding is inconsistent with previous cell culture, animal experiment and autopsy results and may be related to the following: (1) GABA is present in 80% of the striatum, but its concentration is extremely low, and its resonance peak is easily hidden by the peaks of other metabolites. In addition, the sample size of this study is too small, resulting in no difference in the obtained data; (2) the 3.0 T MRI MEGA-PRESS spectrum editing sequence combined with LCModel software has certain limitations in measuring GABA concentrations in vivo. The problem of an unclear peak separation and a low signal-to-noise ratio has not been improved, and the quality of the spectrum still needs to increase. Some other factors may contributed this results, such as small head movements during the scan procedure, relation to akinetic-rigid and tremor dominant expressions.

At present, there are four main ways to improve the quality of the spectrum: (1) increase the magnetic field strength [18]; (2) develop a corresponding pulse sequence and develop a corresponding spectrum editing technology [19–21]; (3) develop the corresponding postprocessing technology [22, 23]; (4) increase the dimensionality of the spectrum. According to Bloch's theorem, increasing the strength of the magnetic field increases the intensity of the measured signal proportionally. However, due to the unknown biological effects produced by strong magnetic fields, the current magnetic resonance machines for clinical applications mostly have field strengths below 3.0 T. Therefore, the idea of relying on the first way to improve the quality of the spectrum is limited. Improving the quality of the spectrum is mainly considered from the latter three aspects.

In this study, the GABA concentration in the striatum was measured by HPLC 3 months after the PD model was established in monkeys. It was found that the GABA concentration in the striatum of the PD monkey was increased and the elevated GABA concentration was positively correlated with the PD monkey behavioral score. However, for the measurement provided by the 3.0 T MEGA-PRESS technique, the GABA concentration on the affected side was not correlated with the behavioral score. It is not difficult to conclude that the GABA concentration obtained by the 3.0 T noninvasive spectroscopy technique has limitations and it has not been confirmed that there is a correlation between the GABA concentration measured by the 3.0 T MEGA-PRESS technique and the behavioral score in PD monkeys. Previously, the authors used this technique to investigate the relationship between the mean striatum GABA concentration, the clinical course of disease, and the unified Parkinson's disease rating scale (UPDRS) score in 45 patients with PD and found no significant correlation between them [24]. Moreover, Bai et al. [25] used the 3.0 T MEGA-PRESS technique to study the correlation between the GABA concentration in the frontal striatum and the degree of cognitive impairment in 15 patients with Alzheimer's disease; they found no significant correlation. However, some scholars used 7 T MRS technology to study patients with mild to moderate PD. The LCModel method was used to find that the GABA content of the putamen and the lesions of PD were slightly correlated, while other metabolites showed no obvious correlation [3]. However, there were only 13 cases of PD in that study. The severity of PD cases was unevenly distributed, and the age difference was large (56 ± 10 years old). The results were not sufficient to verify the GABA concentration in PD patients and the pathological Braak stage. In addition, it is also possible that the changes in GABA levels in PD patients are not synchronized with clinically relevant motor symptoms and disease duration. Evaluating this possibility requires further increasing the sample size and conducting research.

## Conclusions

In conclusion, this study used 3.0 T MRS to measure the concentration of GABA in the striatum of living animals and to compare it with the concentration of GABA in isolated striatum to determine the sensitivity and feasibility of using noninvasive MRS to measure neurotransmitter concentrations in early stages. We found that the elevated levels of GABA in the striatum of PD patients were positively correlated with behavioral changes. Furthermore, the 3.0 T MRS technique can only measure a limited concentration of GABA in vivo, so it is necessary to further increase the magnetic field strength and optimize the editing techniques. The sample size of this study was small; the field strength was 3.0 T and there was no comparison with high field magnetic resonance; thus, a further increase in the sample size and further comparative analysis are required.

## Methods

### Experimental animals

Eight healthy male cynomolgus monkeys weighing 8–10 kg and aged 6–9 years were used. The experimental animals were provided by the Guangxi Xiongsen Primate Experimental Animal Breeding Development Co., Ltd., and the experimental design was were approved by the ethics committee of the First Affiliated Hospital of GuangXi Medical University, China (No. KY-055,2012). In accordance with the feeding requirements for medical experimental animals, feeding was carried out in a single cage, 12 h of circular illumination daily, and sufficient water and fresh fruit were ingested. Six monkeys were PD models (numbered M1 to M6) and 2 were blank control group.

### Establishment of a unilateral PD monkey model by the methyl-phenyl-tetrahydropyridine (MPTP) method

For the cynomolgus monkeys in the PD model group, ketamine (1 mg/kg) intramuscular anesthesia was applied, the exposed common carotid artery was separated, and the external carotid artery was temporarily clamped. MPTP (1.0–1.2 mg/kg) was slowly injected into the Left side the common carotid. There is no anterior communicating artery between the left and right anterior cerebral arteries, and MPTP injected in one carotid artery can not reach the contralateral hemisphere. For the control group, only a neck skin incision was made where the common carotid artery branched into the external carotid artery, and no drugs were injected. All cynomolgus monkeys were injected with ampicillin (0.5 g/monkey) after surgery for 7 days to prevent surgical wound infection.

### Animal behavior observation

#### Behavioral score

According to the monkey Parkinson's syndrome score scale created by Kurlan et al. [26], the movement and behavior of the model monkeys were scored before and 1, 3, 5, 7, 9, and 11 days after the PD model was established. A double-blind process was used, each observation lasted 30 min and the average was taken as the actual score.

#### Apomorphine-induced experiment

Six PD monkeys were given an intramuscular injection of apomorphine (APO, 0.1 mg/kg; dopamine (DA) receptor agonist) at 4, 8, and 12 weeks after the PD model was established. The number of rotations per minute were recorded within 3 h of injection, and the average number of laps was determined.

### MRS data acquisition and processing

Eight monkeys underwent MRS examination before the MPTP model was established and 3 months afterward. The monkeys were fasted for 4 h before the examination and were intramuscularly injected with ketamine + promethazine (7.0 mg/kg, 3.5 mg/kg) to ensure the animals were quiet during the MRS scanning.

**Fig. 1:**
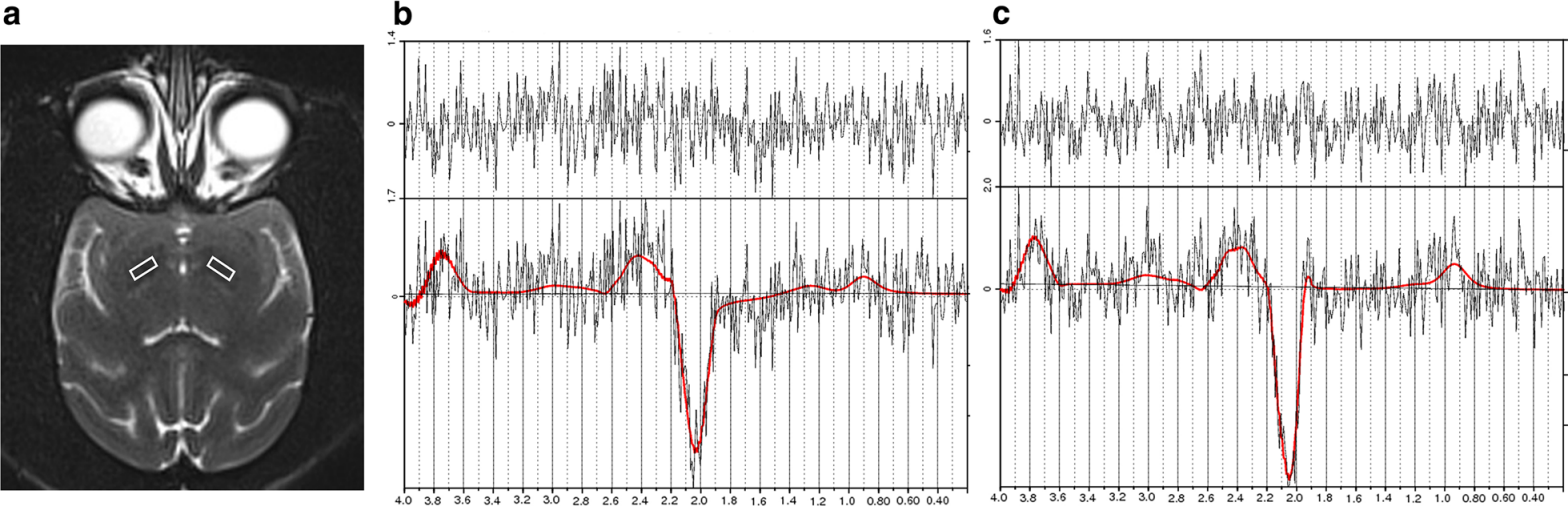
**a** The ROI locations of MEGA-PRESS sequence are shown in the T2-weighted image. **b** LCModel analysis of an edited spectrum was used to determine the GABA content (0.6 µmol/L) at 3.0 ppm in the right striatum of PD monkeys. **c** LCModel analysis of an edited spectrum to determine the GABA content (0.46 µmol/L) at 3.0 ppm in the left striatum of PD monkeys. Upper panels in **b**, **c** are residuals. Lower panels in **b**, **c** are spectrum of LCModel fitting results

Scanning equipment: Siemens 3.0 T magnetic resonance scanner and 12-channel head phased array coil (Siemens, Munich, Germany) was used. The scans for all subjects were completed by the same radiologist. Settings were as follows: 3D prepared rapid acquisition gradient echo (MP-RAGE) sequence localization, scanning range from occipital macropore to the semioval center level, thickness 1 mm, TR 1700 ms, TE 2.9 ms, TI 900 ms, matrix 256 × 256, FOV 220 mm × 220 mm. For the MEGA-PRESS sequence, the region of interest (ROI) was located in the bilateral striatum (Fig. 1a), the leading edge was parallel to the ipsilateral lenticular nucleus, the lateral side was parallel to the lateral edge of the ipsilateral lenticular nucleus and the upper edge was parallel to the upper edge of the ipsilateral lenticular nucleus. Attempts were made to avoid the adjacent cerebrospinal fluid and blood vessels. The ROI size was 20 mm (RL) × 20 mm (AP) × 20 mm (FH) (Fig. 1). First, the spectrum was scanned with the following scan parameters: TR 1500 ms, TE 68 ms, bandwidth 1200 Hz, NEX = 4, Average 128, and WaterSuppresion select saturation. Then, the water peak scan was performed, and the scanning parameters were as follows: TR 1500 ms, TE 68 ms, bandwidth 1200 Hz, NEX = 4, Average 128, and WaterSuppresion select NONE.

**Fig. 2:**
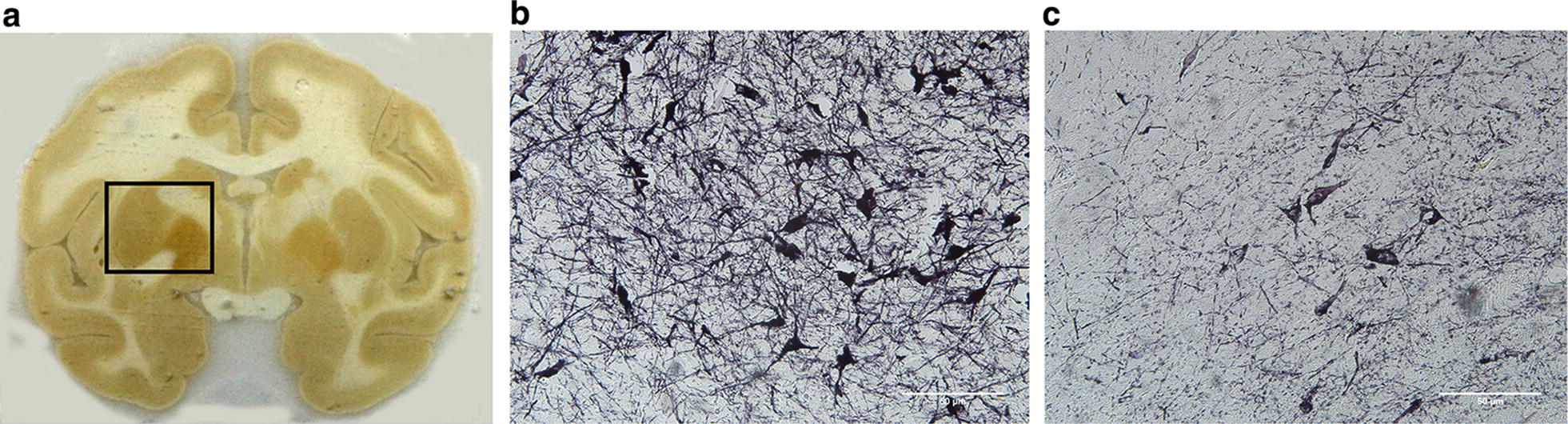
**a** The striatum level of frozen brain sections of PD monkeys. The anatomy of the striatum is shown. **b** The midbrain substantia nigra of the MPTP injection contralateral of the PD monkeys with TH-positive dopaminergic neuron cells (TH Stain, × 20). **c** The midbrain substantia nigra of the MPTP injured side of the PD monkeys (TH Stain, × 20). The number of TH-positive dopaminergic neuron cells in the MPTP injured side was significantly reduced, and the residual nerves denaturated

After the end of the scan, the computer automatically performed the same baseline and phase adjustment for the obtained line to obtain a metabolite frequency distribution curve. The metabolite frequency distribution curve data was then imported into the LCModel postprocessing software package for computational analysis to quantify the concentration of GABA in the striatum (Fig. 2).

### Determination of GABA concentration in isolated brain tissue of experimental animals

After completing the MRS scan test at 12 weeks, monkeys were humanely euthanized by decapitation method in accordance with USAISR IACUC Policy. Briefly, monkeys without analgesics/anesthetics were restrained and were decapitated using a guillotine by a trained person. This method allows to obtain intact brain tissue without chemical contamination. The brain anatomical map of the cynomolgus monkey was compared, and the brain tissue of the same part of the MRS examination was stored (striate nucleus, midbrain), and the frozen tube was immediately placed in liquid nitrogen and stored in a − 70 °C refrigerator for later use. The striatum GABA concentration was measured by HPLC: (1) Preparation of main reagents: (1) stock solution of amino acid standards (L-Glu, L-Gln, GABA) (1.0 mmol/L), stored at -200C. (2) 0.1 M sodium tetraborate (Ph = 9. 6) 100 mL. (3) Mobile phase A: 0.1 mol/L KH_2_PO_4_ (containing 0. 8% THF pH = 5 8.). (4) Derivatization reagent stored at 0–4 °C. (2) Preparation HPLC analysis of the sample supernatant, and the supernatant was taken and eluted according to the chromatographic conditions. (3) Take 5 μL of standard or pretreated sample, adding 25 μL of OPA derivatizing agent for derivatization reaction. The calculation method used is the external standard method, based on the peak area. The formula is as follows: GABA concentration = (A sample/A standard) × standard concentration × standard sample amount × total sample volume/ sample amount × sample weight (unit: μmol/g).

### TH immunohistochemical staining

(1) Brain was fixed in 4% paraformaldehyde. SN sentions (40 mm) were made by using freezing sliding microtome. The SN sections were washed with 0.01 M PBS. (2) The SN sections were blocked with 3% H_2_O_2_ to eliminate endogenous peroxidase. After washed with 0.01 M PBS, the sections were incubated with blocking solution (5% bovine serum albumin in PBS) at room temperature for 1 h. (3) The sections were incubated with anti-TH antibody overnight at 4 °C. The sections were incubated with secondary antibodies for 1 h at room temperature after washed with 0.01 M PBS. (4) Immunoreactivity was visualized by incubated in DAB and counterstained with hematoxylin. Control staining was performed by using PBS as the primary antibodies. (5) TH-positive cells in SNc were detected under a light microscope.

### Statistical analysis

Statistical calculations were performed with SPSS 17.0 software. Two independent sample t tests were used for comparisons between two groups and between two sides within the same group. The correlation between GABA concentration and behavioral score was determined by Pearson's correlation. The measurement data are expressed as the mean ± SD. P < 0.05 was considered statistically significant.

## Data Availability

The datasets used and/or analysed during the current study are available from the corresponding author on reasonable request.

## Abbreviations

GABA: γ-aminobutyric acid
PD: Parkinson's disease
MPTP: methyl-phenyl-tetrahydropyridine
HPLC: high-performance liquid chromatography
PET: positron emission tomography
MRI: magnetic resonance imaging
ROI: region of Interest
GAD: glutamic acid decarboxylase
MRS: magnetic resonance spectroscopy
MPP+: l-methyl-4-phenylpyridinium
SPECT: single-photon emission computerized tomography
NAA: *N*-acetylaspartate
Cr: creatine
Cho: choline
Lac: lactate
MI: myo-inositol
Glu: glutamate
Gln: glutamine
